# Improved production of human type II procollagen in the yeast *Pichia pastoris *in shake flasks by a wireless-controlled fed-batch system

**DOI:** 10.1186/1472-6750-8-33

**Published:** 2008-03-27

**Authors:** Maria Ruottinen, Monika Bollok, Martin Kögler, Antje Neubauer, Mirja Krause, Eija-Riitta Hämäläinen, Johanna Myllyharju, Antti Vasala, Peter Neubauer

**Affiliations:** 1 Bioprocess Engineering Laboratory, Dept. of Process and Environmental Engineering, University of Oulu, P.O.Box 4300, FIN-90014 University of Oulu, Finland; 2 Collagen Research Unit, Department of Medical Biochemistry and Molecular Biology, University of Oulu, P.O.Box 5000, FIN-90014 University of Oulu, Finland; 3 Biocenter Oulu, University of Oulu, FIN-90014 University of Oulu, Finland

## Abstract

**Background:**

Here we describe a new technical solution for optimization of *Pichia pastoris *shake flask cultures with the example of production of stable human type II collagen. Production of recombinant proteins in *P. pastoris *is usually performed by controlling gene expression with the strong AOX1 promoter, which is induced by addition of methanol. Optimization of processes using the AOX1 promoter in *P. pastoris *is generally done in bioreactors by fed-batch fermentation with a controlled continuous addition of methanol for avoiding methanol toxification and carbon/energy starvation. The development of feeding protocols and the study of AOX1-controlled recombinant protein production have been largely made in shake flasks, although shake flasks have very limited possibilities for measurement and control.

**Results:**

By applying on-line pO_2 _monitoring we demonstrate that the widely used pulse feeding of methanol results in long phases of methanol exhaustion and consequently low expression of AOX1 controlled genes. Furthermore, we provide a solution to apply the fed-batch strategy in shake flasks. The presented solution applies a wireless feeding unit which can be flexibly positioned and allows the use of computer-controlled feeding profiles.

By using the human collagen II as an example we show that a quasi-continuous feeding profile, being the simplest way of a fed-batch fermentation, results in a higher production level of human collagen II. Moreover, the product has a higher proteolytic stability compared to control cultures due to the increased expression of human collagen prolyl 4-hydroxylase as monitored by mRNA and protein levels.

**Conclusion:**

The recommended standard protocol for methanol addition in shake flasks using pulse feeding is non-optimal and leads to repeated long phases of methanol starvation. The problem can be solved by applying the fed-batch technology. The presented wireless feeding unit, together with an on-line monitoring system offers a flexible, simple, and low-cost solution for initial optimization of the production in shake flasks which can be performed in parallel. By this way the fed-batch strategy can be applied from the early screening steps also in laboratories which do not have access to high-cost and complicated bioreactor systems.

## Background

The methylotrophic yeast *Pichia pastoris *is a favored yeast species as a host for heterologous protein production (for reviews see [1-6]). *P. pastoris *has the potential for high expression levels, efficient secretion of target proteins, posttranslational modifications, and is easily grown to high cell densities on mineral salt medium in bioreactors. It has been demonstrated that *P. pastoris *is an efficient production system also for very large and complex proteins, such as collagens, which besides the recombinant gene(s) needed for the collagen polypeptide chain(s) needs the parallel expression of two different genes coding for collagen prolyl 4-hydroxylase (C-P4H), an enzyme required for the thermal stability of collagens [7-9].

Although different promoter systems exist for a controlled or continuous expression of heterologous proteins in *P. pastoris*, frequently the strong and tightly controlled promoter of alcohol oxidase 1 (AOX1) is applied. The AOX promoter is induced by methanol [6]. Methanol, aside from being the inducer of the promoter is also a carbon/energy substrate. Generally, in such *P. pastoris *processes, methanol is the only carbon substrate during the production phase of the AOX1-promoter controlled protein. A drawback is the toxicity of methanol. Generally, methanol concentrations above 3.6% inhibit the yeast growth and lead to death [2].

In previous studies for shake flask cultures, the commonly used protocol recommended by Invitrogen [10] for methanol feeding has been modified individually depending on the product. For the expression of proteins in shake flask cultures it is proposed that methanol is added twice per day [11,12]. However, due to the small window in which the methanol concentration must be kept for an optimal process, pulse-based methanol addition for optimization of processes in shake flasks seems not to be the best way to provide data for the development of a fermentation process.

Optimization work for recombinant processes with *P. pastoris *is generally performed in bioreactors by the fed-batch strategy. The basic scheme follows in principle the original protocol of Brierley and coworkers [13]. A glycerol batch phase is followed by a transition phase and by a methanol induction phase [14]. Thereafter, methanol is added continuously. Different variations of this strategy have been proposed, mostly evaluated as being advantageous for a specific protein (comprehensively reviewed by [2]).

The requirement of bioreactors is a limitation for parallel fast optimization of processes. Principally parallel bioreactor systems exist on the market, but they are generally expensive and non-scalable. Here we describe a solution, which allows the application of the fed-batch principle in shake flasks. We apply a recently described monitoring system [15] which is connected to a radio-modem controlled feeding device, which can be placed directly on shakers.

The recombinant protein produced in the present investigation is human type II collagen which is the major collagenous component in the cartilage [16]. Purified recombinant human type II collagen is regarded very useful in applications for cartilage repair [17]. The formation of stable triple helical collagen at physiological temperature requires C-P4H activity [16,18]. Consequently, the coexpression of C-P4H, an α_2_β_2 _tetramer located within the lumen of the endoplasmic reticulum, has been proven essential for the production of temperature-stable recombinant collagens [7,8]. Recombinant type II collagen has been produced successfully in the baculovirus expression system [19,20] and in *P. pastoris *[8,20].

In this study, application of semicontinuous feeding of methanol to shake flask cultures improved significantly the production of stable human type II collagen, which is documented by protein and mRNA analyses of the collagen and C-P4H.

## Results

### pO2 level-dependent manual feeding of methanol improves expression

**Figure 1:**
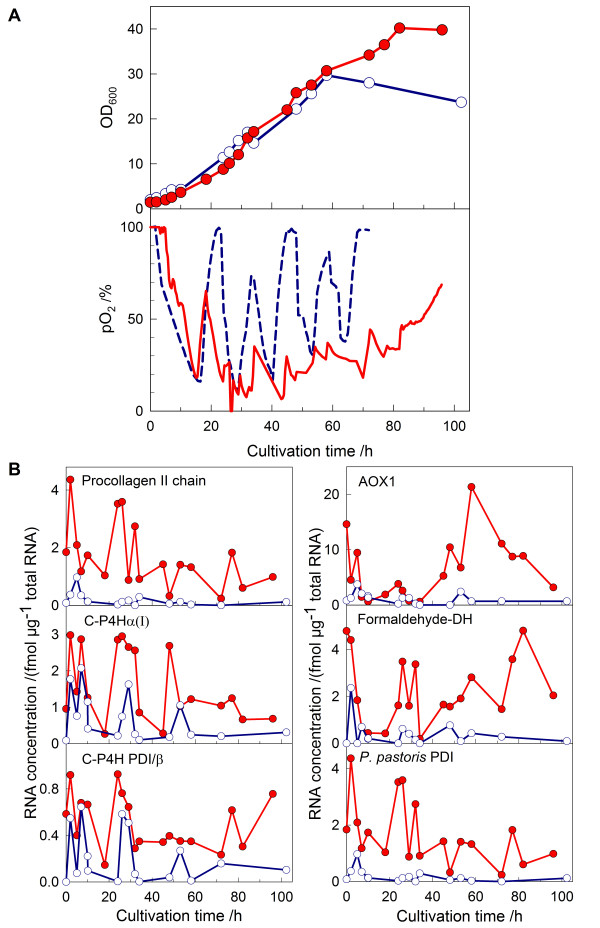
Growth parameters (A) and concentrations of product-related mRNA species (B) during cultivation of a *P. pastoris *for production of recombinant human collagen II in shake flasks. Cultivation procedures with two methanol pulses per day (control, blue open circles, interrupted blue line) and pO_2_-dependent manual feeding of methanol (red filled circles, red continuous line) were compared.

We have shown previously by applying on-line monitoring sensors in shake flasks that the commonly used methanol feeding protocol for shake flask cultures of *P. pastoris *(two manual pulses of methanol per day) leads to long starvation phases between feeding pulses [15]. Starvation is obvious by a pO_2 _peak after exhaustion of methanol, related to declining respiratory activity (Figure 1A). We concluded from this data that the methanol feeding in this shake flask process is not optimal. In the first step of optimization, the rapidly increasing pO_2 _level after consumption of methanol was used as a signal to manually add a new methanol pulse. Each single methanol dose was the same as in the standard protocol and consequently much more methanol was added during the course of the cultivation. Although the procedure was laborious and difficult to reproduce manually, a 30% higher cell density was obtained and the product related mRNAs showed higher expression, such as the mRNAs encoding alcohol oxidase 1 (AOX), formaldehyde dehydrogenase, yeast PDI, type II procollagen chain and the C-P4H α(I) and PDI/β subunits (Figure 1B). Unexpectedly, the amount of collagen II was not increased, however (not shown).

### Quasi-continuous feeding of methanol to shake flasks improves pro(II)collagen production

Although the results from the manual feeding experiment were promising, due to the long cultivation time a real optimization of the feeding procedure would be possible only by a computer-controlled feed, which allows the addition of methanol in a quasi-continuous way, similar as in bioreactor fed-batch cultivations.

**Figure 2:**
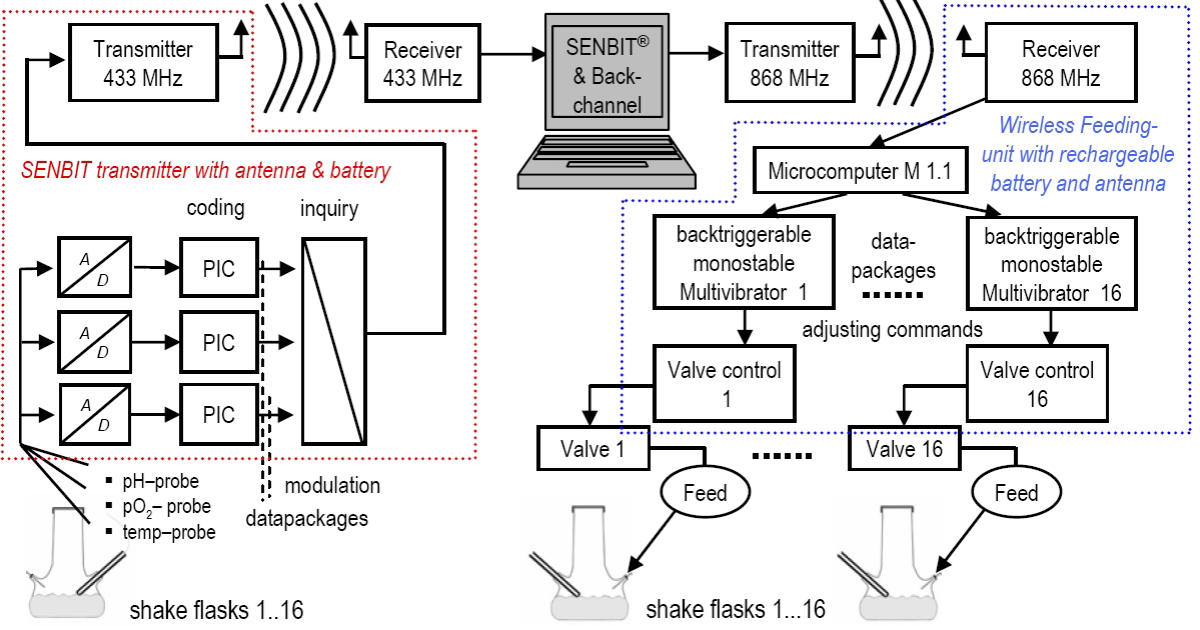
Schematic presentation of the wireless data collection and control system for shake flask cultivations.

Therefore, a computer-controlled feeding device was developed, consisting of an encapsulated microcomputer which was connected to feeding valves. The microcomputer is controlled by a normal desktop computer over a radio-module working at 868 MHz as shown schematically in Figure 2. If needed, the feeding device can be powered by a rechargeable lead battery.

Methanol was fed from a sterile 50 ml syringe through a silicon tube connection (1 mm diameter) and a steel needle connected to one of the three side necks of the shake flask. The feeding pulse was given by a miniature LFV valve (The Lee Company, U.S.A.) which was integrated into the wireless feeding device. In the performed experiments a flow through the valve was simply created by gravitation over an approximately 1 m height difference, but we could also generate a constant flow of solutions with low viscosity by applying an air overpressure container or a micropump (data not shown).

With the developed device and the programmed back-channel software, different feeding protocols could be applied to shake flasks (not shown). Opening of the feeding valves in short time intervals yielded a quasi-continuous flow.

The effect of quasi-continuous methanol feeding into the shake flasks was tested by repeatedly performing series of two parallel 200 mL cultures with different computer controlled feeding schemes.

In each experiment for one of the shake flasks (reference culture) the feeding strategy was based on the commonly used pulse-feeding method [10,12] in which a 5% methanol solution was fed twice a day to a final methanol concentration of 0.5% (v/v). Into the other flasks methanol was fed in a quasi-continuous mode. Within the first 2 hours a methanol pulse was given every 12 min and after that the feeding interval was 30 min. Each methanol pulse was 0.8 ml of the 5% methanol solution. The total feeding volume and total amount of added methanol was the same in both parallel flasks.

**Figure 3:**
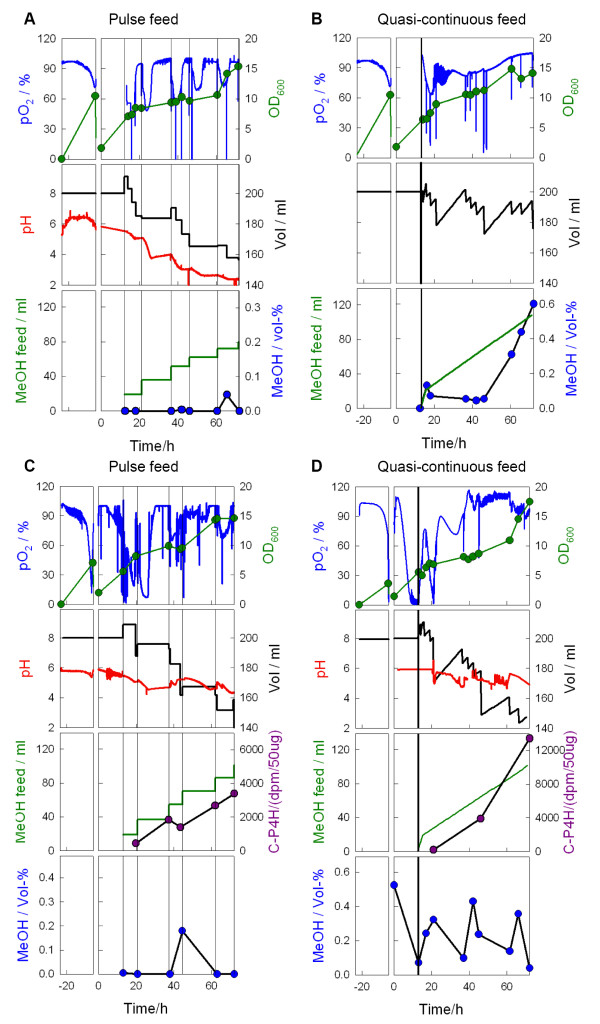
*P. pastoris *cultivation in shake flasks with methanol feeding without (A, B) or with (C, D) manual pH adjustment. Cultivations were performed in two phases: initial batch phase in BMG-medium and fed-batch in BMM-medium. Dissolved oxygen (pO_2_) and pH were measured with a wireless measuring system, cell growth was followed by measurement of the OD_600_. Vertical lines represent methanol feeding points in A and C or start of methanol feed in B and D.

In the reference cultures the addition of a methanol pulse was followed by an immediate drop of the pO_2 _level. However, within 5 to 10 h after a methanol pulse the pO_2 _raised, indicating exhaustion of the substrate (methanol). The pO_2 _dropped to zero only when the shaker was stopped for sampling (see pO_2 _spikes in Figures 3A and 3B), but remained otherwise above 50%.

In the flasks with quasi-continuous feeding the first methanol pulse caused a decrease of the pO_2_. Later, when the feeding intervals were longer, the pO_2 _level was stabilized to about 90% (Figure 3B). Only after 50 h of cultivation, when the methanol started to accumulate, the pO_2 _level increased indicating a higher maintenance or toxification.

The methanol concentration in the culture medium of the reference culture was close to the detection limit almost during the whole incubation time. Only towards the end of the cultivation a low concentration of residual methanol could be detected in the medium. It should be remarked that the samples for methanol analysis were always collected before the next methanol pulse. Therefore we concluded that methanol had been fully consumed before the next pulse. This was also obvious from the pO_2 _profile. In contrast, small amounts of methanol were present in the culture with quasi-continuous methanol addition. Until 50 h of cultivation the methanol concentration was below 1 g L^-1^. Only towards the end of the cultivation an increase of the residual methanol concentration was detected.

As the pO_2 _level was high in the culture with quasi-continuous feeding it was tested whether also a higher feeding rate would be possible. Feeding with a double amount of methanol was not beneficial, but instead led to the accumulation of the methanol concentration above 5 g L^-1 ^already within 20 h after the start of the feeding (data not shown).

Without pH control, the pH value declined during the whole cultivation. The decrease occurred stepwise in the reference culture and was clearly related to the feed pulses (see Figure 3A). At the end of the cultivation the final pH was very low (close to 2), which surely is not beneficial for the cell growth and production. As the pH in fermenter cultivations is usually kept between 4 and 6, we decided to adjust the pH during the experiment to 5.5 by intermittent addition of 10% (v/v) ammonia.

The control of the pH did not result in major changes in the general culture parameters (Figure 3C,D). The variations, especially the higher methanol concentration in the culture with quasi-continuous feed (Figure 3D) may be mainly attributed to the larger amount of samples collected during the cultivation for analysis of the protein product and mRNA levels.

**Figure 4:**
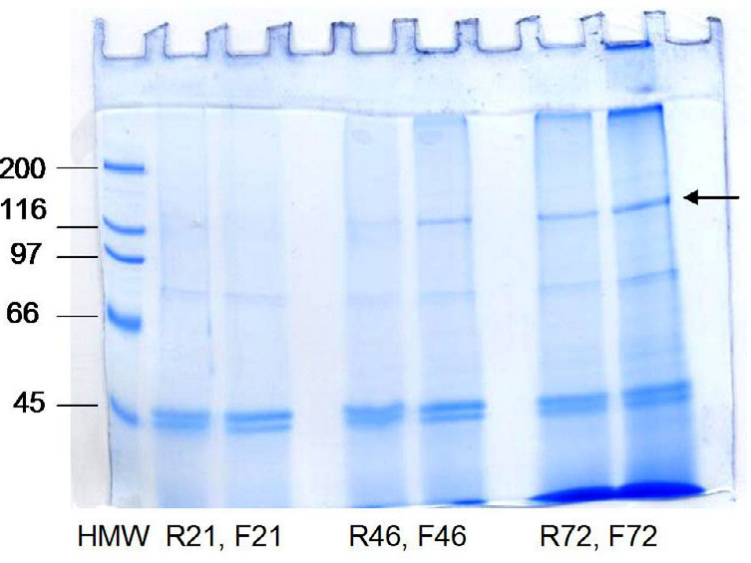
SDS-PAGE analysis of expression of human collagen II in *P. pastoris *shake flask cultivation (experiment 3, Table 1) after 21, 46 and 72 hours cultivation. R represents a reference culture with pulse feeding of methanol and F predetermined quasi-continuous feed. Collagen chains were derived from correctly folded collagen II molecules by HCl-extraction and pepsin digestion. The collagen II chains are marked with an arrow.

All cultures were followed by analysis of collagen II, C-P4H enzymatic activity and product-related mRNAs. The amount of type II collagen chains (139 kD) derived from correctly assembled protease-resistant triple-helical collagen II was analyzed by reducing SDS-PAGE after HCl extraction with pepsin-treatment according to Myllyharju et al. [8]. Stable and correctly assembled collagens are resistant against pepsin. Collagen II showed a clear band (Figure 4) which was significantly stronger in the cultivations with the quasi-continuous feeding profile, compared to the standard pulse feeding profile. The results were reproducible (see Table 1) despite experiment to experiment variations. Generally, more correctly assembled triple-helical collagen was produced in the experiments with quasi-continuous feeding of methanol.

**Table 1:** Review of OD_600_, collagen II expression, C-P4H activities, and the amount of total fed methanol in four independent experiments with parallel predetermined constant feed. The pulse feeding method was used as a reference.

Exp.no.	OD_600 _46/72 h		Pepsin resistant collagen II		C-P4H/(dpm/50 μg protein)		Methanol feed/ml	
	Feed	Reference	Feed	Reference	Feed	Reference	Feed	Reference
1	14.1	15.4	+	++	-	-	109	72
1A	12.2	15.4	+	++	-	-	>100	72
2	11.0/18.7	10.4/14.2	+++	n.d.	15449*	2042*	115	74
3	18.5	17.4	++++	++	13403	1526	85	75
4	19.7	14.6	++	+	6263	3391	104	89
4A	17.8		++		5238		102	

Experiments 1, 2, 3, 4 and 4A have been performed with a quasi-continuous predetermined feeding rate, however in experiment 4A the methanol concentration was doubled compared to the other experiments. Experiment 1A was performed as a pO_2_-stat (computer controlled methanol addition in dependence on the pO_2 _signal). The amount of correctly folded collagen II (pepsin resistant) was evaluated from the SDS-PAGE gels. Highest values of C-P4H activity are shown as dpm per 50 μg of soluble protein. Total methanol feed in the cultivations is shown in the last column.

* 45 h

**Figure 5:**
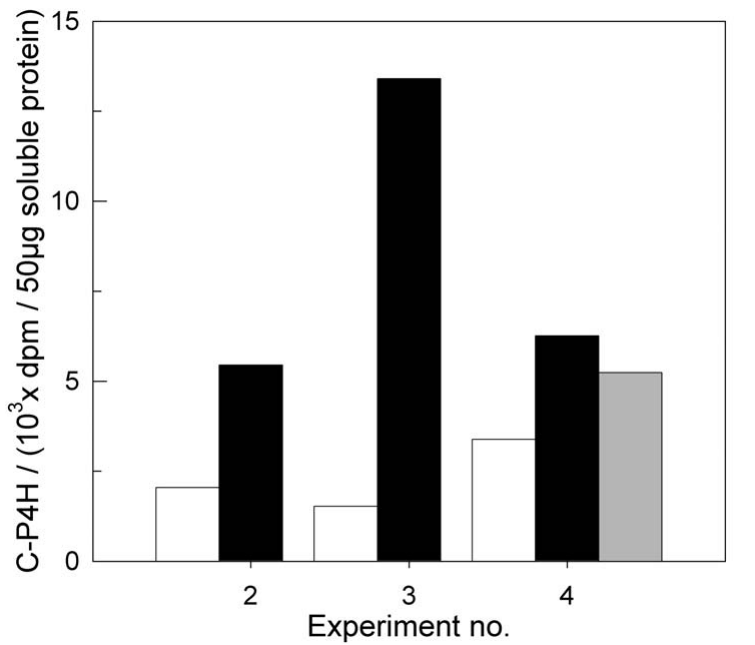
C-P4H activity in three different experiments, each with a quasi-continuous feeding culture (black bars) and a reference culture (manual feeding twice a day, white bars). Additionally, in experiment no. 4 double concentrated methanol was fed (grey bar).

These findings were supported also by the higher amount of C-P4H activity (see Figures 3C and 3D, cf. Table 1). C-P4H activity was measured from several time points and found to be 2 to 8 times higher in different cultures with quasi-continuous methanol feeding compared to the parallel reference cultures in each experiment (see Figure 5). Doubling the methanol concentration in the feeding solution (cf. experiment 4 A in Table 1, and grey bar in Figure 5) led to higher activities compared to the reference, but the C-P4H activity was slightly lower than in the experiment with the lower feed rate. Surprisingly, also the cell density was not increased in this cultivation compared to the culture with quasi-continuous feed of more diluted feed.

**Figure 6:**
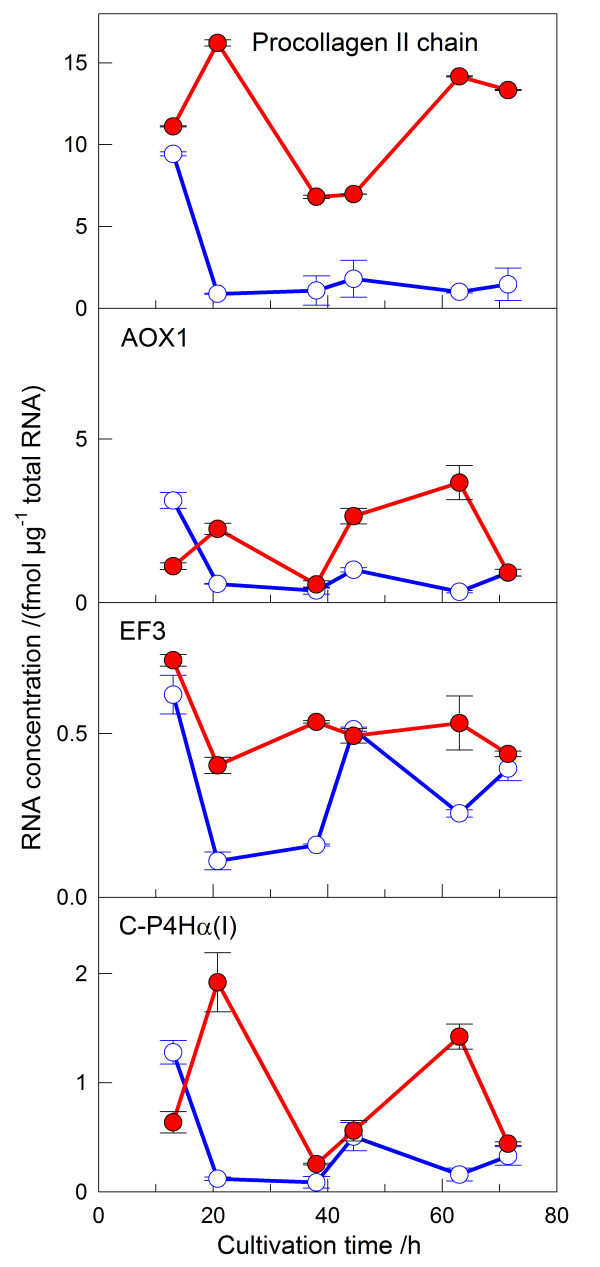
mRNA levels of a) procollagen II chain, b) AOX1, c) translation factor EF3, and d) C-P4H α(I) subunit during shake flask cultivation of *P. pastoris*. Predetermined quasi-continuous feed of methanol was investigated. The cells were first grown in BMG medium and changed to BMM medium at 0 h. The first sample represents the time when the methanol feeding was started (13 h). Predetermined constant feed (red filled circles); reference culture with pulse Feeding (open blue circles). The data are from experiment 4 in Table 1.

The effect of the feeding procedure on the formation of the product was investigated in more detail at the mRNA level. A quantitative sandwich hybridization assay which was developed earlier in our laboratory was applied [21,22]. Figure 6 shows the levels of mRNAs encoding the procollagen II chain, AOX1, EF3 (translational factor), and C-P4H. The level of procollagen II mRNA was already high during the first 12 h when the cultures were grown in a batch mode on methanol in both types of cultivations. In the reference culture with pulse feeding, the procollagen II mRNA level decreased to a very low level after the start of the feeding. In contrast, the mRNA level stayed high during the whole cultivation in the culture with quasi-continuous feeding of methanol, being approximately 10 times higher when compared to the pulse feed method.

The level of AOX1 mRNA, encoding the alcohol oxidase enzyme and therefore indicating the induction of methanol as well as an active methanol metabolism, was much lower than the level of procollagen II mRNA. Interestingly, the procollagen II mRNA decreased fast in the reference culture with pulse feeding strongly during the shift from the methanol batch to the pulse feeding, similarly to all other mRNAs analyzed. Possibly this is a result of growth inhibition due to carbon/energy source starvation as shown above. In contrast, the AOX1 mRNA level was approximately the same over the whole cultivation when the quasi continuous feeding mode was applied. The same behavior was detected for the EF3 mRNA, a translation factor, and for the level of the C-P4Hα(I) mRNA. The higher level of the C-P4Hα(I) mRNA correlates well with the higher amount of C-P4H activity obtained.

## Discussion

Experiments at the scale of shake flasks are generally batch processes. This restricts the applicability of the results for the further development of fermentation processes, which normally apply the fed-batch principle. Despite of this limitation shake flask cultivations are commonly used due to their simplicity and high flexibility.

In many cases it might be useful to apply the fed-batch principle already in the earliest process optimization phase, not primarily for obtaining higher cell densities, but for applying similar physiological conditions as in the later process. As such tools are not commonly available we developed a new feeding system which works wireless and therefore can be easily applied on any shaker. If powered by a lead accumulator, the feeding unit can be directly placed on the shaking table without the need for any direct electrical contact. This unit works together with the earlier described SENBIT wireless measurement system [15]. Control functions and set-points can be easily programmed in the controlling software (BackChannel) which communicates with the measuring software (SENBIT).

Recombinant *P. pastoris *cultures were an interesting test case to apply the feeding system in shake flasks. Especially with the widely used AOX1 promoter system, the results from shake flask experiments may be of low practical value, because the methanol concentration has to be controlled within a narrow concentration window which is not possible in a batch system.

The starting point for our optimization approach was the generally applied recommended protocol for pulse feeding of methanol twice a day [11,12]. This procedure is very different from the various cultivation techniques applied in fermentation processes which all are based on continuous addition of methanol. As model system we used the expression of recombinant human collagen II in *P. pastoris*, which has been described earlier [8]. The assembly of the triple-helical collagen is directly correlated with the hydroxylation of prolyl residues by C-P4H, which is coexpressed together with the collagen polypeptide chains.

It is very obvious that the proposed pulse feeding protocol is unfavorable for collagen production. During cultivation, long phases of carbon/energy starvation between the feeding pulses were observed by measurement of oxygen levels in shake flasks. Presence of these phases was confirmed by analysis of process-related marker mRNAs and analysis of the products. Overall, lower expression of these mRNAs was observed compared to a constant feeding protocol.

We could prove that a quasi-continuous feeding in the shake flask scale can significantly improve the conditions for collagen production and leads to increased expression of triple-helical stable collagen by simultaneous enhancement of C-P4H expression and activity. The corresponding mRNAs were also expressed at higher level than in the culture with pulse feeding.

Almost 10-fold higher amount of procollagen II mRNA was detected in the cultures with quasi-continuous feeding of methanol compared to the pulse method. Also the expression of the translation factor EF3, which is an indicator of protein synthesis, was higher with constant feeding. Generally, in the case of such optimization approaches, and not limited to shake flasks studies, the quantitative analysis of mRNAs, especially for C-P4H and collagen in our case, seems to be a good indicator for the quality of the cultivation.

Although we applied here mainly a predetermined feeding strategy, also other feeding schemes and control principles are applicable with the newly developed feeding system, such as the pO_2_-dependent feeding. Although the DO-Stat control principle did not improve the product yield in our case (data not shown), such extended studies are principally interesting and will widen the usefulness of shake flask cultures.

As discussed above, it is generally not the aim of shake flask studies to reach the high cell densities which are commonly reached in bioreactors. In the experiments described here cell concentrations were rather moderate which is due to the lower oxygen transfer rate in shake flasks compared to bioreactors. It was the intention in our experiments to keep the oxygen level high as it is a necessary cofactor in the C-P4H reaction, although for other proteins good production may be achieved also by running the culture into oxygen limitation which has been recently shown for the production of an scFv antibody fragment [23]. Furthermore, the moderate cell densities in our experiments are also due to the use of diluted methanol feed solutions. To avoid long time intervals without any feed and to ensure a feed which is metabolically seen as continuous methanol was fed as a 5% solution, which correspondingly dilutes the medium and results in a lower final cell yield. The use of low-power micropumps instead of microvalves is currently tested, and may provide an improved continuous supply of small liquid amounts.

## Conclusion

Simple controlled bioreactors can be obtained by applying feeding to shake flask cultivations. Although this will not generally change specific characteristics for shake flask cultures such as the problem to establish specific pO_2 _levels and poorly controllable aeration of the cultures, the proposed solution is a way to control the physiological state of the cultures in early screening phases in a similar way as during later process development. Furthermore, the described system is advantageous in terms of flexibility and investment costs compared to parallel bioreactor systems.

The comparison of the commonly used pulse feeding of methanol to *P. pastoris *cultures in shake flasks with the quasi continuous feeding clearly showed clearly that the commonly applied standard protocol is not favorable to optimize recombinant processes, mainly due to the long-term starvation phases. Commonly such optimization is performed in bioreactors, which however are not available for many of the molecular biology laboratories. We show here a simple solution by applying a quasi-continuous feed profile, which is a method commonly used in bioreactors. However, the developed feeding device is not limited to such simple applications, but pre-determined feed functions and even feed-back control of the feed rate on the basis of measured parameters can be easily programmed and principally turn the shake flask into a bioreactor.

In our case the quasi-continuous feeding profile increased both the amount of C-P4H and stable collagen II. This clearly demonstrates in the case of one complicated large recombinant protein the power of monitoring and control in shaken cultures. The tools applied here provide a valuable system for parallel optimization at small scale also for other kind of cultivations.

## Methods

### Strain

The *P. pastoris *strain sCII7 (Mut^+ ^phenotype) contained the pICZB expression vector with the cDNAs coding for the human procollagen II chain and the two C-P4H subunits (kindly provided from Fibrogen Europe Ltd., Helsinki, Finland). The cells were stored as glycerol stocks at -70°C.

### Cultivation medium and cultivation conditions

*P. pastoris *was cultivated in BMG or BMM culture medium containing 0.1 M phosphate buffer (pH 6.0), 1.34% YNB, 0.4 mg L^-1 ^biotin, and 1% glycerol (BMG) or 0.5% methanol (BMM) respectively [10].

Cultivations were performed in 1 L Erlenmeyer flasks with three baffles and three side necks for the sensors and sampling (Glasgerätebau Ochs GmbH, Bovenden, Germany). The flasks contained 200 mL of BMG and were incubated at 30°C on a rotary shaker (Certomat H, B. Braun Int.) at 250 rpm. When the OD_600 _reached the value between 2 and 6, the cells were centrifuged at 3500 rpm for 5 min, and washed with BMM by further centrifugation. The supernatant was decanted and the cell pellet was resuspended in BMM to obtain an initial OD_600 _of about 1.5. The baffled shake flasks, containing 200 mL of BMM and the pH- and pO_2_-electrodes, were incubated at 30°C on a rotary shaker at 250 rpm. To maintain the expression of the product, 100% of methanol was added to a final concentration of 0.5% (v/v) twice a day or manually at the time when the pO_2 _level increased (shake flask experiment with optimized feeding, cf. Figure 1). Alternatively, the feeding was performed with the wireless feeding device with a 5% or 10% methanol solution as explained in the results section by a quasi-continuous predetermined feed rate, or on the base of the pO_2 _signal (pO-stat principle). In some experiments ammonia (10%) was added manually to keep the pH between 4 and 6, as described in detail in the results section.

#### On-line measurements and control

The SENBIT wireless system (teleBITcom GmbH, Teltow, Germany) was used in the shake flask cultures to follow the pO_2 _and pH as described earlier [15]. The feeding module was constructed as described in the results section.

#### Sampling

The samples were taken with a sterile needle (0.9 × 120 mm) connected to three-way sterile plastic valve with Luer-lock (Oriplast GmbH) inserted into a side neck of the flask and tightened with a rubber seal into 10 ml syringes. After immediate cooling on ice the samples were portioned for the further analyses, with exception for the mRNA samples which were directly chilled in the inhibition solution (see below).

### Analyses

#### Cell growth

Growth was monitored by measuring the turbidity (OD_600_) at 600 nm.

#### Methanol

Samples were centrifuged (2 min, 13000 rpm, 4°C) and the cell-free supernatant was analyzed by gas chromatography.

#### mRNAs

The analysis was performed as recently described [24] with the following modifications for *P. pastoris*. For immediate chilling of metabolic activities *P. pastoris *samples (4 × 2 mL) were immediately mixed with 200 μL inhibition solution (95:5 v/v ethanol/phenol, pre-cooled at -20°C). After centrifugation (2 min, 13000 rpm, 4°C) the supernatant was removed and the pellet was dispensed in 100 μl of RNA Later (Ambion) and stored at -70°C until analysis. Total RNA extraction was performed with the RNeasy Mini Kit and mechanical cell disruption according to the manufacturers instructions (Qiagen).

Oligonucleotide probes (Table 2) were designed using the CloneManager5 program with following submission of the sequences a NCBI BLAST search [25] to exclude alignments with other genes. HPLC-purified unlabelled and biotin-labeled oligonucleotide capture probes were purchased from Metabion GmbH (Martinsried, Germany). Dig-tail labeling of the detection probes was performed according to manufacturer's instructions using the Roche Dig-tailing kit (2^nd ^generation, Roche Diagnostics GmbH, Mannheim, Germany).

**Table 2:** Probes used in RNA sandwich hybridization. The capture probes were labeled with biotin and the detection probes contained a digoxigenin (DIG) tail.

**Probe name**	**Sequence (5**'**-3**'**)**	**Position/Modification**
***human collagen II***		
Helper probe	GCTCCTGGTGATCCTTCTCTA	2918c
Capture probe	CTCTACCTGGAGGACCATCG	2938c [5' biotin]
Detection probe	CTTTAACTCCGGCAGCACCAT	2959c [3' DIG tail]
***C-P4H ***α ***subunit***		
Helper probe	TCTGCTTCTGTATAGGCCAC	536c
Capture probe	TGTTCCATCCACAGTTCCGT	566c [5' biotin]
Detection probe	ATCTCGCCTTCATCCAGTTG	596c [3' DIG tail]
***C-P4H PDI***/β ***subunit***		
Helper probe	CTTGAAGCTCTCGGCTGCTG	849c
Capture probe	TGGTCGCTGTCGATGAAGATG	884c [5' biotin]
Detection probe	TCGAGGATGCGCTGGTTGTC	908c [3' DIG tail]
***AOX1 gene***		
Helper probe	GACTCGTACTGAGGCTTG	1440c
Capture probe	GAATCTCAGCATCACCAC	1475c [5' biotin]
Detection probe	ACCATTGGCGTACCATTG	1510c [3' DIG tail]
***EF3 gene***		
Helper probe	GAATTGACCGGAAGCCAA	1269c
Capture probe	AGCCTCCTTCATATCGAC	1251c [5' biotin]
Detection probe	CTCAGCAACCATCTTGGA	1233c [3' DIG tail]

*In vitro *RNA standards were designed for the quantitative analysis of each gene as described before [22,24]. Therefore, primers as indicated in Table 3 were used for *in vitro *transcription (purchased from Sigma-Genosys, Cambridge, UK).

**Table 3:** Primers for the synthesis of *in vitro *RNA standards. The T7 promoter sequence CTAATACGACTCACTATAGGGAGA was added to the 5'-end of all primer 1 sequences.

**Probe name**	**Sequence (5**'**-3**'**)**	**Position**
***Human collagen II***		
Primer 1	GCCAGGTAGAGAAGGATCAC	2892
Primer 2	CTGCTGCCTCGTCCAAGTAA	4006c
***C-P4H ***α ***subunit***		
Primer 1	GGCAGAAGAGGACAAGTTAG	141
Primer 2	ACAGGCTGCATGCCGTGTA	1509c
***C-P4H PDI***/β ***subunit***		
Primer 1	AAGTACCTGCTGGTGGAGTT	124
Primer 2	TCATCGTCTTCCTCCATGTC	1499c
***AOX1 gene***		
Primer 1	AGCAGGTGAGAACAACCTCAAC	583
Primer 2	AAGGTCCACCGTAGGCATTAGA	1988c
***EF3 gene***		
Primer 1	CGAGTTCGTCAAGCGTGTTC	546
Primer 2	CTCGTCCAGGACGATCAAGT	1434

#### Protein analysis

*P. pastoris *cell pellets were broken with zirconia beads both in acidic conditions followed by pepsin digestion for collagen II analysis. Protein samples were analyzed on SDS-page.

C-P4H activity was analyzed from *P. pastoris *cell pellets that were broken with zirconia beads in basic conditions. The activity assay is based on the hydroxylation-coupled decarboxylation of 2-oxo [1-^14^C] glutarate with (Pro-Pro-Gly)_10 _as the peptide substrate [26]. The total protein concentrations were determined with the RC DC Protein assay Kit (Biorad Laboratories).

## Authors' contributions

MR and MB performed the shake flask experiments. MK and AV developed the wireless feeding system and the computer control program. MB designed the mRNA probes and primers and performed together with MK the mRNA analyses. MR, AN and ERH performed the protein analyses. JM and ERH contributed with their experiences in the collagen production process. MR wrote the initial manuscript, which was read and approved by all authors. PN initiated and practically supervised the scientific work.
